# Association between Frequency of Chromosomal Aberrations and Cancer Risk Is Not Influenced by Genetic Polymorphisms in *GSTM1* and *GSTT1*

**DOI:** 10.1289/ehp.11769

**Published:** 2008-09-01

**Authors:** Anna Maria Rossi, Inger-Lise Hansteen, Camilla Furu Skjelbred, Michela Ballardin, Valentina Maggini, Elena Murgia, Antonio Tomei, Paolo Viarengo, Lisbeth E. Knudsen, Roberto Barale, Hannu Norppa, Stefano Bonassi

**Affiliations:** 1 Department of Biology, Pisa University, Pisa, Italy; 2 Department of Laboratory Medicine, Section of Medical Genetics, Telemark Hospital, Skien, Norway; 3 Department of Aerospace Engineering, University of Naples Federico II, Naples, Italy; 4 Environmental Health, Institute of Public Health, University of Copenhagen, Copenhagen, Denmark; 5 New Technologies and Risks, Work Environment Development, Finnish Institute of Occupational Health, Helsinki, Finland; 6 Unit of Molecular Epidemiology, National Cancer Research Institute, Genoa, Italy

**Keywords:** Bayesian, biomarker, cancer risk, case-control study, chromosomal aberration, genetic polymorphism, glutathione *S*-transferase, *GSTM1*, *GSTT1*, Monte Carlo Markov Chain

## Abstract

**Background:**

The frequency of chromosomal aberrations (CA) in peripheral blood lymphocytes of healthy individuals has been associated with cancer risk. It is presently unclear whether this association is influenced by individual susceptibility factors such as genetic polymorphisms of xenobiotic-metabolizing enzymes.

**Objectives:**

To evaluate the role of polymorphisms in glutathione *S-*transferase (GST) M1 (*GSTM1*) and theta 1 (*GSTT1*) as effect modifiers of the association between CA and cancer risk.

**Methods:**

A case–control study was performed pooling data from cytogenetic studies carried out in 1974–1995 in three laboratories in Italy, Norway, and Denmark. A total of 107 cancer cases were retrieved from national registries and matched to 291 controls. The subjects were classified as low, medium, and high by tertile of CA frequency. The data were analyzed by setting up a Bayesian model that included prior information about cancer risk by CA frequency.

**Results:**

The association between CA frequency and cancer risk was confirmed [OR_medium_ (odds ratio)_medium_ = 1.5, 95% credibility interval (CrI), 0.9–2.5; OR_high_ = 2.8, 95% CrI, 1.6–4.6], whereas no effect of the genetic polymorphism was observed. A much stronger association was seen in the Italian subset (OR_high_= 9.4, 95% CrI, 2.6–28.0), which was characterized by a lower technical variability of the cytogenetic analysis. CA level was particularly associated with cancer of the respiratory tract (OR_high_= 6.2, 95% CrI, 1.5–20.0), the genitourinary tract (OR_high_ = 4.0, 95% CrI, 1.4–10.0), and the digestive tract (OR_high_ = 2.8, 95% CrI, 1.2–5.8).

**Conclusions:**

Despite the small size of the study groups, our results substantiate the cancer risk predictivity of CA frequency, ruling against a strong modifying effect of *GSTM1* and *GSTT1* polymorphisms.

During the last few decades, evidence concerning the role of chromosomal aberrations (CA) in carcinogenesis has been enriched by a number of epidemiologic studies showing that high CA frequencies in peripheral blood lymphocytes of healthy individuals are associated with increased cancer risk (Boffetta et al. 2007; Bonassi et al. 1995, 2000, 2004; Brøgger et al. 1990; Hagmar et al. 1994, 1998, 2004; Liou et al. 1999; Rossner et al. 2005). A case–control study nested within ESCH (European Study Group of Cytogenetic Biomarkers and Health) cohort studies (Bonassi et al. 1995; Hagmar et al. 1994, 1998) found that the strength of such association is not influenced by occupational exposure to carcinogens or tobacco smoking (Bonassi et al. 2000). Contrasting results were reported by other investigations, such as a cohort study on Czech workers (Smerhovsky et al. 2002) describing a stronger association between CA frequency and cancer incidence in miners exposed to radon. Whether the CA–cancer association reflects (occasionally undetected) exposure to carcinogens, individual susceptibility to carcinogens, some form of chromosomal instability, or a causal role of chromosomal rearrangements in the carcinogenic process is still an open issue.

A striking amount of data supports the hypothesis that individual characteristics associated with cancer risk, such as inherited differences in metabolic enzymes or DNA repair capacity, may also modify CA occurrence (Norppa 2004). These findings raise the obvious question of whether the association between CA and cancer risk depends on individual metabolism and DNA repair capability, so that CA would better predict cancer risk in people with an unfavorable genotype. This question can be assessed by incorporating genotype data to the CA–cancer studies, but such an approach has not been applied previously, as DNA samples have not been readily available.

Among the most frequently studied polymorphisms are those concerning the metabolism of xenobiotics, in particular glutathione *S*-transferases (GSTs). GSTs catalyze the conjugation between glutathione and reactive xenobiotic compounds in a pathway that leads to thioethers excreted in the urine (Bolt and Thier 2006; Parl 2005). The major biological function of GSTs is considered to be protection against electrophilic chemical species, although metabolic activation involving GST-mediated glutathione conjugation has also been described (e.g., for some chlorinated substrates) (Bolt and Thier 2006; Parl 2005). Detoxification by glutathione conjugation can represent a minor (e.g., styrene oxide) or a major (e.g., benzo[*a*]pyrene) metabolic pathway for many genotoxic agents. Given the importance of GSTs in the detoxification of electrophilic carcinogens, the possible influence of polymorphisms in *GST* genes on cancer risk has been investigated extensively (Bolufer et al. 2006; Bolt and Thier 2006; Carlsten et al. 2008; Hiyama et al. 2008; Parl 2005; Shi et al. 2008; Vineis et al. 2007; White et al. 2008). The GST M1 (*GSTM1;* BC036805, GenBank) and theta 1 (*GSTT1*; X79389, GenBank) genes have received much attention because of the high prevalence of homozygous deletions resulting in null genotypes with a decreased ability to detoxify carcinogenic compounds, placing null individuals at increased cancer risk (Bolufer et al. 2006; Bolt and Their 2006; Hiyama et al. 2008; Parl 2005; Shi et al. 2008; White et al. 2008).

The availability in the ESCH database of a group of subjects genotyped for *GSTM1* and *GSTT1* genes in three national laboratories allowed us to address the question if these genetic polymorphisms can modify the strength of the association between CA and cancer risk. In a group of subjects from Pisa, Italy—not included in the original ESCH database—cytogenetic slides could be retrieved from freezers, stained, and scored all together, minimizing the impact of protocol variation, reagents drift, and scorer heterogeneity.

The small size of study groups is a common limitation of biomarker validation studies. The presence of sparse data and missing covariates are conditions for which the Bayesian paradigm presents some advantages over the frequentist paradigm. In particular, Bayes’ rule opens the possibility of improving the estimation of the parameters of interest by including the prior probability, based on knowledge already available—in this case, the association of CA with cancer risk.

This study aimed at *a*) confirming the presence of the association between CA and cancer risk in a partially new data set, and *b*) evaluating the role of *GSTM1* and *GSTT1* polymorphisms as effect modifiers of this association. Ancillary but still important aims were to evaluate the impact of reduced laboratory/technical variability on risk estimates and to verify whether Bayesian statistics can provide reliable estimates in small strata.

## Material and Methods

### Subjects

A nested case–control study was performed pooling data from cytogenetic surveillance studies carried out from 1974 to 1995 at the Department of Biology, University of Pisa, Pisa, Italy; the Department of Occupational Medicine, Telemark Hospital, Skien, Norway; and the Danish National Institute of Occupational Health, Copenhagen, Denmark. These laboratories analyzed *GSTM1* and *GSTT1* polymorphisms in subjects screened for CA and followed up in the framework of the ESCH (Bonassi et al. 2000; Hagmar et al. 1998, 2004) and the more recent CancerRiskBiomarkers (Cytogenetic Biomarkers and Human Cancer Risk) (Norppa et al. 2006) collaborative projects.

The Italian cohort consisted of 1,650 healthy subjects selected from the general population living in three areas in Tuscany (Pisa, Cascina, and Navacchio) enrolled between 1991 and 1993 (Barale et al. 1998a, 1998b; Landi et al. 1999; Milillo et al. 1996). The Norwegian cohort included 681 healthy subjects, either occupationally exposed or referents, collected between 1974 and 1990 (Brøgger et al. 1990; Hansteen et al. 1978, 1984). The Danish data arose from a biomonitoring study conducted in 1987 in a group of 226 male stainless steel welders and referents (Knudsen et al. 1992). Information was gathered on demographic, occupational, and lifestyle factors at the time of blood sampling.

In the Nordic countries, all malignant tumors diagnosed from the date of CA testing until the end of the follow-up (2006 in Norway, and 2003 in Denmark) were retrieved through linkage from national cancer registries. In Italy, the cause of death was obtained from the municipality of residence (2006). At the end of the follow-up, a total of 107 cancer cases with information about *GST* polymorphism were tracked (105/107 cases genotyped for *GSTM1* and 77/107 for *GSTT1*). Within each national cohort, controls were matched with their corresponding case by sex, age (± 10 years), and year of CA test (± 5 years). The overall cases to controls ratio was around 1:3 (107 cases/291 controls).

In all participating laboratories, the standard cytogenetic protocol was applied using heparinized whole blood and harvesting the cells after 48 hr of culture (Buckton and Evans 1973). CA preparations were stained with Giemsa. In Italy, the slides were banked at −20°C at the time of the original cytogenetic study (1991–1993) and stained in Giemsa immediately before the new CA analysis. In Italy, 150 and in Denmark, 100 metaphases per donor were scored for CA by a single scorer. In Norway, 100 or (in more recent studies) 200 cells per subject were scored by the same three microscopists. Savage’s classification criteria for CA were used (Savage 1976). Total CA was defined as the number of cells with aberrations, excluding gaps, per 100 cells. To standardize for interlaboratory variation, all subjects were classified as low (1–33 percentile), medium (34–66 percentile), or high (67–100 percentile) according to tertiles of CA frequency distribution. More details on the laboratory protocols can be found in previously published reports (Barale et al. 1998a, 1998b; Bonassi et al. 2000; Hagmar et al. 1994, 1998, 2004; Hansteen et al. 1978, 1984; Knudsen et al. 1992; Landi et al. 1999; Milillo et al. 1996). The study protocols were approved by the ethics committees and legal authorities in each country.

### DNA extraction and genotype analysis

In Italy, genomic DNA was extracted from frozen whole blood or serum as described by Murgia et al. (2007). In Norway, DNA was extracted from fixed cell suspensions with the method described by Skjelbred et al. (2006). When cell suspensions were not available, DNA was extracted from unstained slides kept at room temperature for 3–26 years. The slides were hydrated, dried carefully before adding 100 μL buffer (50 mM Tris, 1 mM EDTA, 0.5% Triton 100), covered with plastic film, kept for 15 min at 56°C, scraped in 50–100 μL dH_2_O 2–4 times (total volume 200 μL), and centrifuged at 3,000 × *g* for 5 min; 50 μL buffer (as above) and 5 μL proteinase K (20 mg/mL) were added to the pellet. The samples were kept on a Thermomixer (Eppendorf, Hamburg, Germany) at 56°C, 1,000 rpm for 3 hr, before deactivating the enzyme on the Thermomixer at 96°C, 1,000 rpm for 10 min. The suspensions were stored at −20°C. The success rate depended upon the amount of cells, the age of the slides, and the genotype tested. In Denmark, DNA from mononuclear blood cells, originally analyzed for DNA repair by the unscheduled DNA synthesis technique and stored frozen in 0.4 M phosphate buffer in 1987, was used for the genotyping. This DNA had been isolated by using Millipore (Bedford, MA, USA) Microcon YM-30 centrifugal filter devices.

In Italy, genotype analysis was performed by a specific multiplex *GSTT1*/*GSTM1* PCR assay as described by Murgia et al. (2007). In Norway, polymorphisms of the *GSTM1* and *GSTT1* (and *GSTP1*) genes were analyzed simultaneously by multiplex polymerase chain reaction using primers described by Nedelcheva Kristensen et al. (1998). A detailed report of the methods used for genotyping the Danish samples can be found in Ko et al. (2000).

### Statistical analysis

As the response variable in the study was binary (cancer yes/no), we used the standard logistic regression approach in which the response variable is the log odds (or logit). The purpose was to estimate the probability that the *i*th person will develop a cancer, conditionally on the information about his/her CA frequency *E_i_* [1 = L (low), 2 = M (medium), 3 = H (high)], age *A_i_*, sex *E_i_*, and smoking habit *S_i_*, which are the design variables (M1):


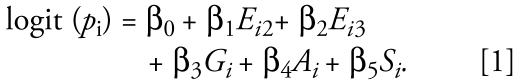


To perform the analysis, we used the Gibbs sampler, which is a Monte Carlo Markov Chain (MCMC) sampling algorithm. MCMC is a class of methods for sampling from probability distributions based on constructing Markov chains. The iterative procedure simulates a Markov chain, which has the desired posterior distribution as its stationary distribution.

We used the statistical software WinBUGS (http://www.mrc-bsu.cam.ac.uk) (Lunn et al. 2000) to set up a Bayesian model specifying an informative normal prior distribution for the regression coefficients where existing knowledge was available. More precisely, from the pooled cohort analysis of 22,358 subjects examined for CAs in 11 countries (Bonassi et al. 2008), we knew that the overall odds ratio (OR) for the medium tertile compared with the low was 1.3 with a 95% confidence interval (CI) of 1.07–1.60, and that the OR for the highest tertile was 1.4 (1.16–1.72). The assumption that the log OR is normally distributed leads to a normal distribution with mean value given by ln(1.3) = 0.26 and ln(1.4) = 0.33, respectively. The standard deviation of the normal distributions was found by solving the equations 1n(1.3) + 1.96**σ** = 1n(1.60) and 1n(1.4) + 1.96 **σ** = 1n(1.72), which gave a standard deviation on the log odds scale of 0.1 in both cases. As suggested by Birkes and Dodge (1993) and Gelman et al. (1995), a simple way to provide a prior variance on the parameters **β**_1_ and **β**_2_ in the current study was to inflate the historical variance. To take into account the heterogeneity and other differences between current and historical data, we considered the prior variance as a Gamma random variable which, on average, fluctuated around the inflating factor (60 in our case). Further prior information was obtained from International Agency for Research on Cancer (IARC) monograph on tobacco smoking (IARC 1986): (weighted) mean value of OR (current vs. never smokers) was considered 10 for lung cancer, 1.4 for oral cavity**–**digestive tract cancers, and 2.1 for genitourinary organ cancers. With regard to the Bayesian interval estimation of OR, we adopted the 95% credibility interval (CrI) with the highest posterior density, that is, an interval of the OR posterior distribution such that the area under the curve is 0.95 and the density at any point inside the interval is greater than the density at any point outside. The traditional CI based on frequentist inference refers to repeated sampling, random intervals, and true parameter values (OR) and does not provide any estimates concerning the study that was actually conducted. On the contrary, CrI is a probability statement within the data set studied concerning the random variable OR and specifying the range within which the OR lies with 0.95 probability.

To estimate if the *GSTM1* null and *GSTT1* null genotypes were a risk factor for all cancers, we used a similar multivariate analysis, adding to the model M1 described in Equation 1 the variables *GSTM1*, *GSTT1* (we called this model M2) and their interactions within CA (we called this model M3). The analysis performed to choose between competing models was based on the BIC index, *BIC* = −21n*L_m_* + *m*1n*n*, where *n* is the sample size, *m* is the difference of the number of parameters in the models, and *L_m_* is the likelihood ratio for the models. This criterion penalized models that improved the fit, increasing the number of parameters. The model with the lower value of BIC was the one to be preferred.

## Results

The distribution of cases and controls in the whole database by country and by other variables considered in the analysis is given in Table 1. The proportion of males and of current smokers was higher in the databases from Norway and Denmark. Smoking habit was not different between the cases and controls. The Italian subjects selected from the general population had a higher mean age, a higher proportion of women, and fewer current smokers than the Nordic groups. The distribution by tumor site of the 107 cancer cases included in the study is reported in Table 2.

The distribution of *GST* polymorphisms did not differ significantly in the three countries, and therefore all data were combined before analysis (Table 3). No significant differences were evident in the genotype distribution of cases and controls, with null genotype prevalence of 52.4 and 50.5% for *GSTM1* and 29.9 and 29.8% for *GSTT1*, respectively. Accordingly, the mean frequency of CA did not appear to be modulated by genotype (Table 3).

The univariate analysis comparing the overall mean CA frequency (SE) in cases and controls [1.98 (0.14) vs. 1.38 (0.14)] revealed a highly significant difference (*t*-test, *p* = 0.006). At the national level, cancer cases had a significantly higher mean CA frequency in the Danish and Italian databases (data not shown).

The results of the multivariate Bayesian model linking CA tertile and cancer risk are reported in Table 4. No effect of sex or age were observed; smoking habit increased the overall risk of cancer but did not confound or modify the effect of CA level on cancer risk. The overall analysis showed a borderline risk increase for subjects in the medium tertile of CA distribution (OR = 1.5; 95% CrI, 0.88–2.50) and an increase for those in the high tertile (OR = 2.8; 95% CrI, 1.6–4.6) when compared with the lowest tertile. Increased cancer risks for the medium and high tertiles were found in all national data sets, although only in the highest tertile of the Italian subset did the CrI not include 1 (OR = 9.4; 95% CrI, 2.6–28.0).

The possible role of *GSTM1* and *GSTT1* genetic polymorphisms as effect modifiers of the association between CA frequency and cancer risk was tested in the Bayesian models (model M3 vs. model M2). The analysis, based on the BIC index, favored the model without interaction terms (M2), ruling against the hypothesis that the cancer risk predictivity of CA frequency could be modified by these polymorphisms, i.e., BIC(M3) = 481.9 > BIC(M2) = 473.1 for *GSTM1* and BIC(M3) = 365.5 > BIC(M2) = 357.7 for *GSTT1.*

A similar multivariate model was used to estimate if the *GSTM1* null and *GSTT1* null genotypes were a risk factor for all cancers (model M2 vs. model M1). Again, the BIC index did not show any increase in the null genotypes, as anticipated by the results of the univariate analysis, i.e., BIC(M2) = 473.1 > BIC(M1) = 467.3 for *GSTM1* and BIC(M2) = 357.7 > BIC(M1) = 352.6 for *GSTT1.*

Finally, we fitted multivariate Bayesian models to major cancer sites to test the hypothesis that CA frequency could more specifically predict the risk by cancer type (Table 5). Credible associations (see statistical methods for a definition of credibility intervals) were found with the high tertile of the CA distribution for cancers of the genitourinary tract (OR = 4.0; 95% CrI, 1.4–10.0); respiratory tract (OR = 6.2; 95% CrI, 1.5–20.0), and digestive tract (OR = 2.8; 95% CrI, 1.2–5.8).

## Discussion

The results of this case–control study provide new information for the validation of CA as a biomarker of cancer risk. Although the study groups were rather small, our findings suggest that the association between CA and cancer is not modified by *GSTM1* and *GSTT1* polymorphisms, that the association is higher when the evaluation is based on uniform cytogenetic data rather than pooled historical data, and that the use of Bayesian modeling is a credible approach for risk estimation in case of sparse data, a common condition in biomarker validation studies.

Our study offered a rare opportunity to assess the impact of genetic polymorphisms on the relationship between chromosomal damage and cancer risk with a longitudinal design. We were able to evaluate only two genotypes, that is *GSTM1* and *GSTT1*, because of the limited amount of DNA available for each subject. Previous data have suggested that the null genotypes of both *GSTM1* and *GSTT1* are associated with a slightly increased risk of some forms of cancer, although this does not appear to concern all cancers (Bolufer et al. 2006; Bolt and Thier 2006; Carlsten et al. 2008; Hiyama et al. 2008; Parl 2005; Shi et al. 2008; Vineis et al. 2007; White et al. 2008). There is also evidence in favor of *GSTM1* and *GSTT1* genotypes affecting the level of CA in lymphocytes especially in smokers, although most studies have suggested no clear effects of either polymorphism on the baseline level of CA (Norppa 2004; Tuimala et al. 2004; Vodicka et al. 2004). Furthermore, the possible presence of heterogeneity in the genetic background of national populations, as well as the activation of carcinogens occasionally caused by polymorphic *GST*s (Hu et al. 2006; Kligerman and Hu 2007), may have weakened the associations studied. Thus, our negative findings on the modifying effect of the two *GST* polymorphisms on the association between CA and cancer risk seem to agree with the existing information about their negligible influence on total cancer risk and baseline CA level. Our conclusions are, however, limited by the small size of the study groups, which did not allow assessing possible interactions between genotype and smoking. Moreover, the genotype data were obtained using different sources of DNA, and thereby different methods, for DNA isolation and genotyping in the participating laboratories.

In general, the present findings confirmed, largely with new data, the results published by the ESCH in 2000 (Bonassi et al. 2000)—that cancer prediction based on CA frequency is independent of smoking and exposure to carcinogens.

The polymorphisms of other genes, such as those involved in DNA repair, might have been relevant as well (Tuimala et al. 2004; Vodicka et al. 2004). However, polymorphisms in the DNA repair genes *OGG1* (8-oxoguanine DNA glycosylase; U96710, GeneBank), *XRCC1* (X-ray repair complementing defective repair in Chinese hamster cells 1; M36089, GeneBank), *XRCC3* (X-ray repair complementing defective repair in Chinese hamster cells 3; AF035586, GeneBank), *ERCC2* (exicision repair cross-complementing rodent repair deficiency, complementation group 2; HGNC:3434, GeneBank), and the folate metabolism gene *MTHFR* (5,10-methylenetetrahydrofolate reductase; BC053509, GeneBank) were evaluated in the Norwegian data set (Skjelbred et al. 2006) and explained only 4–10% of the variance in CA, suggesting that these polymorphisms would not have strong effects on the association between CA frequency and cancer risk. Although many genetic polymorphisms have been observed to affect the level of chromosome damage (Norppa 2004; Skjelbred et al. 2006; Tuimala et al. 2004; Vodicka et al. 2004), single or a few genes may be expected to have only a small effect on CA frequency.

Most studies of CA and cancer risk have been multicentric, and the cytogenetic data generated during many years in a number of laboratories are subject to methodologic variability due to multiple scorers and differences in cell culture, slide preparation, staining, and analysis. A unique feature of the current Italian data was that the cytogenetic slides of the cancer cases and selected controls, originating from surveillance studies performed in Pisa in the early 1990s (Barale et al. 1998a, 1998b; Landi et al. 1999; Milillo et al. 1996), were retrieved from the freezer, stained, and consecutively scored for CA by a single microscopist specifically for the present study. Most probably, such procedure reduced technical variability, and this is the most likely explanation for the very high risk estimates in Pisa, previously reported only by another case–control study from Taiwan (Liou et al. 1999). This observation raises an interesting point concerning the use of CA as a biomarker of cancer risk. The cancer risk associated with a high frequency of CA, although consistently detected in all studies thus far published (Boffetta et al. 2007; Bonassi et al. 1995, 2000, 2004; Brøgger et al. 1990; Hagmar et al. 1994, 1998, 2004; Liou et al. 1999; Rossner et al. 2005), has generally been low and variable. Therefore, it has been considered that CA can hardly be applied for individual risk assessment. If the findings of this study are confirmed, and a stronger association between CA frequency and cancer risk can be obtained by reducing technical variability, the perspective for the use of this biomarker in cancer prevention may have to be rethought. Uniform CA analysis is a routine practice in cytogenetic surveillance studies, which are mostly interpreted at the group level, with the exception of radiation biodosimetry. For group-level evaluation, the present practice of scoring 100–200 cells per person is adequate, but it is likely not sufficient for reliable individual CA assessment. However, if the level of CA had, in reality, a stronger association with cancer risk than has thus far been assumed, reasonable individual cancer risk estimation might become feasible with extended CA analysis. At any rate, our findings lend further support to earlier considerations (Bonassi et al. 2005; Norppa et al. 2006) that the CA assay could be used more widely in estimating cancer risk at the group level.

Another innovative feature of the present study was the potential provided by the Bayesian approach for improving the reliability of the estimates based on data from small groups, as often happens with biomarker validation studies. Most recent results from large CA cohort studies, such as the Czech cohort (Rossner et al. 2005) and the new cohort of Central and Eastern European countries (Boffetta et al. 2007), described stronger associations with specific cancers. In smaller cohorts and in case–control studies, risk estimates based on classic frequentist modeling are likely to produce unreliable estimates in small strata, and any likelihood-based analysis for small data, or even worse, missing data, usually involves computationally intensive methods or *ad hoc* adjustments. Even without epidemiologic biases, the presence of small counts indicates that large statistical biases may affect the point estimates (Greenland et al. 2000).

In contrast, the Bayesian analysis allows one to efficiently manage extra information, sparse data, and missing covariates. In the present study, MCMC sampling enabled us to make inferences for any sample size without resorting to asymptotic calculations. In many cases, a frequentist inference can be obtained as a special case of the Bayesian inference, and when vague prior information is used, point and interval estimates are similar to the frequentist counterpart. However, the fundamental difference is the interpretation of intervals of variability associated with risk estimates. This is the best possible description of risk variability within the study context. If the study is correctly designed and the prior information is correctly selected, this risk range provides the most credible assessment of risk variability.

Interestingly, the present results showed the strongest associations of CA with the same cancer types reported by the cohort studies mentioned above, that is, cancers of the digestive (Boffetta et al. 2007; Rossner et al. 2005) and respiratory tract (Bonassi et al. 1995), although the numbers of specific cancer cases in the present evaluation were small.

In conclusion, this nested case–control study provided relevant new information for interpreting the role of chromosomal damage in carcinogenesis. In particular, it suggested that reducing technical variability in the cytogenetic analysis may increase the strength of the association between CA and cancer risk, with possible implications for the results provided by cohort studies published so far, which were affected by large discrepancies in laboratory protocols and scoring. The negative result concerning the influence of *GSTM1* and *GSTT1* polymorphisms, despite the intrinsic limitation due to small numbers, is in agreement with the idea that individual polymorphisms are not expected to have a dramatic influence on baseline CA level or overall cancer risk. Our findings support the hypothesis that CA frequency, although indirectly measured in surrogate tissues, can predict the risk of cancer by itself as a phenotypic manifestation of multiple carcinogenic processes or as an intermediate step of a causal process. This conclusion is of great value for the use of CA as biomarker in cancer prevention policies.

## Figures and Tables

**Table 1:** Number of cancer cases and controls classified according to sex, age, and smoking status.

	Italy		Norway		Denmark		Total	
Group	Case/control	Total	Case/control	Total	Case/control	Total	Case/control	Total (%)
Sex								
Male	21/48	69	64/165	229	12/49	61	97/262	359 (90.2)
Female	8/23	31	2/6	8	0/0	0	10/29	39 (9.8)
Age (years)								
≤ 39	0/0	0	13/44	57	5/16	21	18/60	78 (19.6)
40–47	2/5	7	12/41	53	1/19	20	15/65	80 (20.1)
48–55	1/13	14	20/39	59	4/10	14	25/62	87 (21.9)
56–61	9/20	29	7/34	41	2/4	6	18/58	76 (19.1)
≥ 62	17/33	50	14/13	27	0/0	0	31/46	77 (19.3)
Smoking^a^								
Never	13/28	41	25/61	86	4/22	26	42/111	153 (38.4)
Former	9/23	32	7/21	28	5/19	24	21/63	84 (21.1)
Current	7/20	27	33/81	114	3/8	11	43/109	152 (38.2)
Total	29/71	100	66/171	237	12/49	61	107/291	398 (100)

a At the time of CA sampling. For one case and eight controls from Norway, no data were available on smoking status.

**Table 2:** Distribution of cancer cases included in the nested case–control study by site and country.

	No. of cases				
Tumor site (ICD–9 code)^a^	Italy	Norway	Denmark	Total	Percent of all cases
Oral cavity (140–149)	0	1	1	2	1.9
Esophagus (150)	1	0	0	1	0.9
Stomach (151)	1	3	0	4	3.7
Intestine, colon, and rectum (152–154)	4	8	1	13	12.1
Liver (155)	3	2	0	5	4.7
Pancreas (157)	4	1	0	5	4.7
Larynx (161)	0	1	0	1	0.9
Lung (162)	6	5	1	12	11.2
Bone, skin (170–173)	0	19	1	20	18.7
Breast (174)	2	0	0	2	1.9
Uterus (179,182)	1	0	0	1	0.9
Ovary (183)	0	1	0	1	0.9
Prostate (185)	1	6	0	7	6.5
Bladder (188)	2	4	1	7	6.5
Kidney (189)	1	2	0	3	2.8
Other sites	3	13	7	23	21.5
Total	29	66	12	107	100.0

a International Classification of Diseases, 9th Revision (WHO 1975).

**Table 3:** Distribution of *GSTM1* and *GSTT1* genotypes in cases and controls and mean CA frequency.^a^

	Total			Mean CA %			
Genotype	Cases (%)	Controls (%)	Total (%)	Cases (SE)	Controls (SE)	Total (SE)	Two-sample Kolmogorov–Smirnov test of identical distribution functions (null vs. positive)
*GSTM1*							
Null	55 (52.4)	141 (50.5)	196 (51)	2.13 (0.2)	1.42 (0.1)	1.62 (0.1)	*p* = 0.69
Positive	50 (47.6)	138 (49.5)	188 (49)	1.83 (0.2)	1.47 (0.1)	1.57 (0.1)	
All	105	279	384				
*GSTT1*							
Null	23 (29.9)	65 (29.8)	88 (29.8)	1.78 (0.36)	1.20 (0.15)	1.35 (0.2)	*p* = 0.67
Positive	54 (70.1)	153 (70.2)	207 (70.2)	1.80 (0.17)	1.46 (0.12)	1.55 (0.1)	
All	77	218	295				

a Because of the limited amount of DNA retrieved from stored specimen, only 105/107 cases were genotyped for *GSTM1* and 77/107 for *GSTT1*.

**Table 4:** Multivariate Bayesian estimates of cancer risk by tertiles of CA frequency by country.^a^

	Italy		Norway		Denmark		Total	
CA level	Case/control	OR (95% CrI)	Case/control	OR (95% CrI)	Case/control	OR (95% CrI)	Case/Control	OR (95% CrI)
Low	4/40	1.00	22/73	1.00	6/27	1.00	32/140	1.00
Medium	8/19	2.9 (0.8–8.3)	22/58	1.3 (0.7–2.3)	1/10	1.0 (0.2–2.7)	31/87	1.5 (0.9–2.5)
High	17/12	9.4 (2.6–28.0)	22/40	1.9 (1.0–3.4)	5/12	2.0 (0.6–5.1)	44/64	2.8 (1.6–4.6)
Total	29/71		66/171		12/49		107/291	

a Estimates based on 20,000 (MCMC) updates.

**Table 5:** Multivariate Bayesian risk estimates of cancer risk by tertiles of CA frequency and by cancer site.

Cancer site (ICD–9 code)		
CA tertile	Case/control	OR (95% CrI)
Oral cavity–digestive tract (140–159)		
Low	9/140	1.00
Medium	8/87	1.4 (0.6–2.8)
High	13/64	2.8 (1.2–5.8)
Larynx/lung (160–169)		
Low	1/140	1.00
Medium	4/87	2.2 (0.6–6.1)
High	8/64	6.2 (1.5–20.0)
Genitourinary organs/bladder (179–189)		
Low	4/140	1.00
Medium	5/87	1.6 (0.5–3.8)
High	10/64	4.0 (1.4–10.0)
